# *In Silico* Assessment for Risk of Possible Human Transmission of FCoV-23

**DOI:** 10.1155/2024/8398470

**Published:** 2024-10-01

**Authors:** Ahmet Caglar Ozketen, Hasan Huseyin Kazan, Cenk Serhan Özverel, Tamer Şanlıdağ

**Affiliations:** ^1^ DESAM Research Institute Near East University, Nicosia, Cyprus; ^2^ Department of Medical Genetics Faculty of Medicine Near East University, Nicosia, Cyprus; ^3^ Genetics and Cancer Diagnosis-Research Center Near East University, Nicosia, Cyprus

**Keywords:** coronaviruses, Cyprus, feline, *in silico* modeling

## Abstract

Since the pandemic in 2019, coronaviruses (CoVs) have been a great concern for public health burden. The fact that CoVs can infect all animals including domestic ones and livestock points to a future pandemic even though interaction between human and wildlife animals is restricted. Moreover, interspecies transmission abilities of CoVs by mutations make them drastically risky not only for humans but also for animal health. Recently, a new CoV outbreak in cats in Cyprus, the so-called FCoV-23, has been realized. In addition to worries over animal health, any possible transmission to humans is now controversial. However, there have been limited characterization studies on FCoV-23. Thus, we aimed to assess the possible transmission of FCoV-23 to humans using *in silico* prediction tools. Accordingly, we first checked the binding affinities of receptor binding domain (RBD) of FCoV-23 against feline target protein and its human homolog. Next, we randomly and rationally created mutations on the RBD sequence and evaluated the binding affinities using protein docking tools. Our results underlined that multiple mutations at the same time were needed for increased binding affinity towards human target protein, demonstrating that the probability of transmission to humans was extremely low when mutation rates were regarded. Still, further molecular studies are required to comprehensively conclude the possible transmission risk.

## 1. Introduction

Coronaviruses (CoVs) are the family of positive strand RNA viruses known as Coronaviridae. They are notorious for being the causative agent of severe respiratory and digestive diseases in animals including severe acute respiratory syndrome (SARS), Middle East respiratory syndrome (MERS), and feline infectious peritonitis (FIP). Due to the devastating impact of the previous two epidemics (SARS-CoV-1 and MERS-CoV) and a recent pandemic (SARS-CoV-2), the primary focus has shifted to human-associated CoVs [1, 2].

Cross-species transmission abilities of CoVs increase the concerns about these viruses [3]. However, the presence of intermediate hosts is required for many cases of transmission such as palm civets for SARS-CoV-1, dromedary camels for MERS-CoV, and pangolins for SARS-CoV-2 [4]. Even transmissions from humans to cats and dogs have been reported, emphasizing the magnitude of cross-species transmission [5].

Recently, a new outbreak of CoV (FCoV-23) has been realized in the cats in Cyprus [6]. Feline coronaviruses (FCoVs) present a major threat to cats since minor cases of FCoV-associated enteritis may develop into FIP [7]. FCoVs could be categorized into two groups based on their genome; feline-originated FCoV-1 and FCoV-2, an FCoV-1 biotype recombined with canine coronavirus (CCoV) on spike (S) region and nearby 3a, 3b, and 3c genes [8]. Alterations in the S protein critically affect the host–virus interaction via modulation of interaction pattern of the receptor binding domain (RBD) with the target host protein, which may bring about cross-species transmission [9].

According to the nature of the CoVs, there is an urge to monitor and characterize FCoV-23 to understand and predict future outcomes. In the present study, we evaluated the risk of FCoV-23 to interact with human amino-peptidase 1 (hAPN), the homologous of the feline amino-peptidase 1 (fAPN), target protein of the FCoV-23 in feline, via *in silico* estimations. We analyzed the S proteins by comparison to their closest relatives to answer the question of what is required to switch the receptor of choice from fAPN to hAPN. We randomly created mutations in the RBD amino acid sequence of FCoV-23. Then, by employing protein–protein interaction and protein docking tools, we estimated the alterations required for FCoV-23 to threaten the human population. Our *in silico* predictions underlined that multiple mutations on FCoV-23 are needed for binding to the hAPN. However, when the low mutation rate of CoVs is considered, the probability of transmission is regarded not to be alarming.

## 2. Materials and Methods

### 2.1. Conserved Domain Analysis

FCoV-SB22, FCoV-UG-FH8, FCoV-23, porcine respiratory coronavirus (PRCV), transmissible gastroenteritis virus (TGEV), FIPV, human coronavirus 229E (HCoV-229E), and NL63 (HCoV-NL63) were used to study conserved domains architecture among spike proteins. Spike protein sequences of seven alphacoronaviruses (FCoV-SB22, FCoV-UG-FH8, PRCV, TGEV, FIPV, HCoV-229E, and NL63) were retrieved from the NCBI virus database, whereas FCoV-23 was obtained from Atippa et al. [6]. All spike proteins were annotated against conserved domain database (CDD) to predict domain architecture [10]. Batch CD-search web tool was used with the *E* value threshold (expect value) of 0.0001 to search for best fits [11].

### 2.2. Multiple Sequence Alignment and Phylogenetic Tree Analysis

Clustal omega (ClustalW) web tool from the EMBL-EBI (The European Bioinformatics Institute) website was employed on the eight sequences of alphacoronavirus to align amino acid sequences [12]. Only predicted RBD of spike proteins were chosen to generate multiple sequence alignment. The output was analyzed using the BioEdit tool [13] and uploaded to the Interactive Tree of Life (iTOL v6) tool to construct phylogenetic tree [14]. The output was presented with branches of different colors to emphasize the relationship.

### 2.3. Protein–Protein Interaction Analysis and Protein Docking

In the present study, primary or tertiary structures of the proteins, hAPN, fAPN, FCoV-23 RBD, CCOV-HuPn-2018 RBD, and HCoV-229E RBD, were used depending on the requirement of the *in silico* prediction tools. CCOV-HuPn-2018 RBD and HCoV-229E RBD, whose binding affinities have already been shown for fAPN and hAPN, respectively, were chosen as the controls of the *in silico* predictions. The primary structures that were obtained from the NCBI database of the hAPN and fAPN, and FCoV-23 RBD whose sequences were taken from article by Atippa et al. [6] were given in the Supporting Information 1: File S1. For the tertiary structures, the already studied ones were directly obtained from the Protein Data Bank (PDB; RCSB.org; [15]). The PDB codes for the proteins were 6U7G ([16, 17]chain A for hAPN; 6U7G chain C for HCoV-229E RBD; 7U0L [18, 19] chain A for fAPN; and 7U0L chain B for CCoV-HuPn-2018 RBD). For FCoV-23 RBD, the PDB file created by Atippa et al. [6] was directly used.

### 2.4. Prediction of Protein–Protein Interactions and Protein Docking

Using the primary structure of the proteins, we determined the binding sites of the proteins by iFrag server [20]. Next, we predicted the protein–protein interaction using the tertiary structures of the proteins by CCharPPI server [21] and docking by ClusPro 2.0 [22].

### 2.5. Mutation Testing

To create random mutations on the FCoV-23 RBD amino acid sequence, we used the Mutate Protein option of the Sequence Manipulation Suite tool (https://www.bioinformatics.org/sms2/mutate_protein.html; [23]). By random mutations, we created 9 different FCoV-23 RBD sequences where single (mut1-3; Supporting Information 2: File S2), triple (mut4-6; Supporting Information 2: File S2), and quinary (mut7-9; Supporting Information 2: File S2) amino acids were altered when blasted to the original sequence by NCBI BLAST tool [24]. In addition to those random mutations, we rationalized to create exact two mutations on the interaction regions of the FCoV-23 RBD, and one of them was deletion (mut10;Supporting Information 2: File S2) and deletion plus quinary mutations (mut11; Supporting Information 2: File S2). The tertiary structures of the mutated structures were modeled by SWISS-MODEL [25], and all predictions were conducted using the tools given above.

## 3. Results and Discussion

The Coronavirus disease (COVID-19) pandemic caused by the transmission of SARS-CoV-2 to humans has resulted in great concerns about the CoV family for possible future pandemics [26] owing to the critical variants of the virus [27] and copandemic potential [28]. After this pandemic, the interaction between humans and animals, evolutionary hosts of the CoVs have been questioned [29]; nevertheless, CoVs are able to infect a huge variety of species such as wildlife, domestic animals, and livestock [30–32] which makes it difficult to limit the interaction of human with animals within the global ecosystem [33]. Moreover, well-documented cross-species transmission phenomena should be taken into remarkable consideration for future pandemic scenarios [4, 34–36].

Recently, a new CoV outbreak has been reported in the cats of Cyprus, called FCoV-23. FCoV-23 has caused FIP with numerous possible cat deaths through Cyprus Island, threatening other countries with domestic cat transports. Although it has been an emergent issue on the island, there have been limited studies reporting the characteristics of the virus [6]. Beyond the animal losses that endanger the ecological balance on the island, the question of possible transmission to humans remains unclear. Thus, we tried to answer this question using *in silico* predictions.

**Figure 1:**
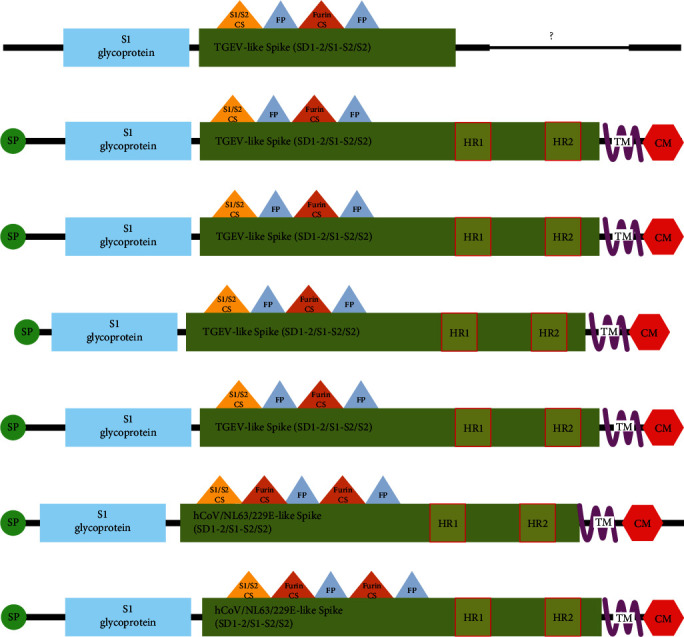
Conserved domain analysis on members of alphacoronavirus. S1: S1 glycoprotein domain(pfam01600) of coronavirus, conserved TGEV-like_Spike_SD1-2_S1-S2_S2: domain (cd22377) located on C-terminal S1 region and S2 region of spike proteins, conserved HCoV/NL63/229E-like_Spike_SD1-2_S1-S2_S2domain (cl40439): located on C-terminal S1 region and S2 region of spike proteins, HR: heptad repeat, TM:transmembrane domain, CM: cysteine-rich C terminal intravirion (cl41189) motif, SP: signal Peptide, CS:cleavage site, and FP: fusion peptide. (A) FCoV-23, (B) FIPV, (C) FCoV-SB22 AND FCoV-UG-FH8, (D) PRCV, (E) TGEV, (F) HCoV-229E, and (G) HCoV-NL63.

First, we checked the domain conservation between other CoVs. FCoVs are members of the alphacoronavirus genus also including FIPV, FCoV-SB22, FCoV-UG-FH8, PRCV, TGEV, HCoV-229E, and HCoV-NL63 [7]. Using the limited sequence information submitted by the article of [6], we compared the domains of known alphacoronavirus members (Figure 1). All spike proteins began with secretion signal peptide followed by the S1 region. The S1 region was homologous to TGEV-like S1 in all spikes except for HCoV-229E and NL63. Correspondingly, protease cleavage sites and fusion peptide domains were similar except for HCoV-229E and NL63 signifying their close relativeness with each other in terms of conserved domain architecture. Accordingly, depending on the length and position of the S1 glycoprotein and TGEV-like spike domains, FCoV-23 was closer to FIPV, FCoV-SB22, FCoV-UG-FH8, and TEGV. Nonetheless, we could not evaluate the heptad repeat, transmembrane domain, and cysteine-rich C terminal intravirion motif owing to the lack of the protein sequence of FCoV-23.

**Figure 2:**
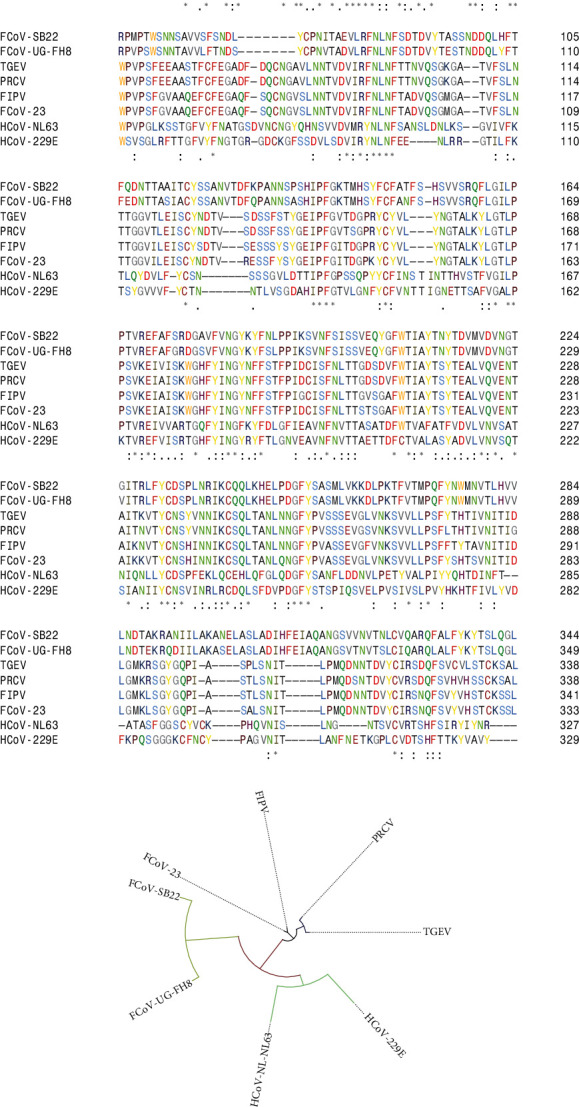
Relationship between different alphacoronaviruses in terms of RBD region. (A) Multiple sequence alignment analysis and (B) schematic representation of phylogenetic analysis.

The interaction between the host receptor and spike protein of CoV mainly depends on RBD domains [37]. Therefore, it is an effective strategy to compare the sequences of RBD of different alphacoronaviruses interacting with different host receptors. The RBD domains of the eight CoVs have significant homology with each other (Figure 2A). The relation between RBD regions is in parallel with conserved domain architectures. FCoV-23 closely resembles FIPV, moderately to other FCoVs, and remotely to HCoVs. The phylogenetic tree was constructed with these results summarizing the relationship between each other (Figure 2B).

**Figure 3:**
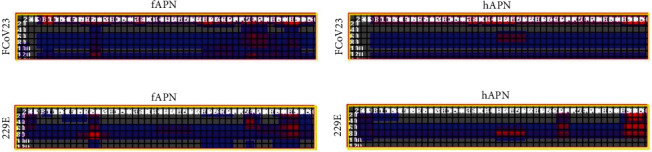
Primary structure-based interaction profiles of the proteins. Heat maps for the interaction of (A) FCoV-23 RBD-fAPN, (B) FCoV-23 RBD-hAPN, (C) HCoV-229E RBD-fAPN, and (D) HCoV-229E RBD-hAPN. Gray: The regions that are not involved in the interaction. Blue to red: Weak to strong interaction regions.

After conservation analyses, we predicted the protein–protein interactions using the iFRAG server. Based on the amino acid sequences of the proteins, fAPN, hAPN, FCoV-23 RBD, and HCoV-229E RBD, we predicted their possible interaction regions [20]. According to the results, mainly the sequences in the middle and the C terminus of the FCoV-23 RBD interacted mostly with the C terminus of the fAPN protein (Figure 3A). However, these interaction patterns were not realized in FCoV-23 RBD-hAPN counterparts (Figure 3B). When interactions of HCoV-229E binding to hAPN [16] were investigated, the heat maps for the C terminus of fAPN (Figure 3C) and hAPN (Figure 3D) were drastically different from each other, pointing out the diversity of fAPN and hAPN proteins whose BLAST result confirmed highly altered amino acid sequences (Supporting Information 2: File S2). This result underlined that not only the primary structure of the CoV RBDs but also that of the host protein itself was critical for the interaction pattern.

Next, we investigated the interactions of fAPN, hAPN, FCoV-23 RBD, and HCoV-229E RBD using the CCharPPI server. This server requires tertiary structures of the proteins to predict the interactions by several tools depending on the Gibbs free energy calculations [21]. Among these tools, we chose AP_Complex [38], ZRANK [39], RosettaDock [40], and pyDock [41] tools for the assessment of the interaction between the proteins. According to the results (Table 1), fAPN but not hAPN was predicted to interact with FCoV-23 RBD, whereas HCoV-229E RBD resulted in a reverse situation. This result showed that FCoV-23 by the known amino acid sequence cannot interact with hAPN.

To further investigate any possible interaction by potential mutations in the FCoV-23 RBD, we randomly created nine different mutations as three sets of singular, triple, and quinary alterations (Supporting Information 2: File S2). Moreover, regarding the interacting regions of the HCoV-229E RBD sequence, we rationally created two more mutations by deletions (Supporting Information 2: File S2). Next, we predicted the possible interactions between the mutated FCoV-23 RBD with fAPN and hAPN using iFRAG and CCharPPI servers by the primary and modeled tertiary structures, respectively. According to the iFRAG results, the interacting regions between hAPN and mutated sequences did not match with that of HCoV-229E and hAPN (data not shown), and the CCharPPI server was not efficient in discriminating the prediction of interactions (Supporting Information 3: File S3). Thus, we used another protein docking tool, ClusPro. Depending on the high-rank conformation, the Gibbs free energies calculated by ClusPro designate the interaction capabilities of the proteins [22, 42]. Accordingly, we predicted the possible interactions between hAPN and FCoV-23 RBD, HCoV-229E RBD as control, and mutated FCoV-23 RBDs. The results indicated lowest energy was desirable for the interaction of hAPN and HCoV-229E RBD, whereas this value was higher for hAPN-FCoV-23 RBD (Table 2). Amongst the mutated RBDs, the possible interaction between hAPN and only mut8 gave the lowest energy, underlying that by this mutation set, FCoV-23 could bind to hAPN. In the set of mut8, there are five different alterations compared to the original FCoV-23 RBD sequence (Supporting Information 2: File S2). Two of the amino acids were replaced with charged residues, namely glutamic acid (E) and lysine (K). The remaining three-point mutations were aromatic amino acids tryptophan (W) and tyrosine (Y) replacements in mut8. Charged and aromatic amino acids are indispensable to establish protein receptor interactions such as cation–*π* interactions [43]. Their presence or absence could determine electrostatic interactions and hydration capacity which would ultimately adjust transmissibility and infectivity [44–46]. In a similar pattern, aromatic amino acids are detrimental in terms of receptor binding by interacting with sugar moieties such as sialic acid [47, 48]. Contrary to our expectation, the mutations were positioned outside of the interaction site and still drastically changed the binding energy. However, when we modeled mut8 and compared it with wild-type RBD of F-CoV-23, we observed slight structural differences on *β*-loops of *β*-sheet regions (Supporting Information 3: File S3). Hence, it is possible that the increase in binding affinity may be a result of these slight changes. This issue was also reported for the binding profile of the SARS-CoV-2 *δ* variant B.1.617.2 [49], confirming the effect of the *β*-loops of *β*-sheet regions on the binding affinities.

Depending on the mutation rate, the probability of five exact mutations at the same time is extremely low [50]; however, fewer mutations may increase the possibility. Therefore, after deciphering a mutation set with five amino acid alterations putatively binding to hAPN, we intentionally decreased the mutated amino acids to find out whether fewer mutations were enough to induce affinity against hAPN. Particularly focusing on the charged amino acids, we eliminated the number of mutations in the mut8, created four novel mutation sets (mut8_1, mut8_2, mut8_3, and mut8_4; Supporting Information 2: File S2), and predicted the binding affinities against hAPN using ClusPro 2.0 tool. According to the results, mut8_4 displayed higher affinity against hAPN (Table 3) compared to the affinity of wild-type FCoV-23 RBD (Table 2). Importantly, not the charged amino acids but the aromatic ones were more influential in the increased affinity.

Finally, we tried to illuminate the mutation rates of FCoV-23 to model the probability of multiple mutations at the same time. The large genome of CoVs (26–32 kb) is subjected to changes by means of recombination events and mutations [51]. However, CoVs have comparatively low rate of mutations (1 × 10^−3^ substitutions per base per year (sub/nuc/yr); Table 4) compared to other viruses such as poliovirus (1 × 10^−2^ sub/nuc/yr) [53] as the moderate fidelity is due to the activity of nonstructural protein 14 (nsp14) to provide further proofreading to RNA polymerase (nsp12) action [54, 55]. Overall, due to the fact that human betacoronaviruses have a higher potential to be zoonotic [33] in addition to the low mutation rate of CoVs, FCoV-23 has a limited potential to transmit to humans.

## 4. Conclusion

In the present study, we investigated the possible transmission of FCoV-23, a new CoV infecting cats in Cyprus, to humans by *in silico* predictions. We showed that characterized FCoV-23 cannot interact with human target protein; thus, the transmission would be not possible. Nonetheless, we emphasized that multiple mutations in the RBD of FCoV-23 could result in increased affinity towards the human target protein, making the transmission possible. However, when the mutation rate and subtype of FCoV-23 are considered, we propose that the probability of transmission is extremely low. Still, further molecular studies are necessitated and human–animal should be minimal during this outbreak.

## Figures and Tables

**Table 1:** Prediction of protein–protein interactions using CCharPPI server.

Proteins	AP_DComplex	ZRANK	RosettaDock	pyDock	Interaction
hAPN-FCoV 23 RBD	−4.7	−0.23	0.008	0.406	No
fAPN-FCoV 23 RBD	−12.00	−74.41	−5.99	−30.61	Yes
hAPN-HCoV 229E RBD	−11.11	−76.93	−7.41	−31.06	Yes
fAPN-HCoV 229E RBD	6.35	98.18	801.26	17.95	No

**Table 2:** Prediction of protein–protein interactions using the ClusPro 2.0 tool.

Proteins	Structure	Members	Weighted score center (kcal/mol)	Weighted score lowest energy (kcal/mol)
hAPN-FCoV23	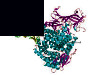	97	−846.8	−846.8
hAPN-229E	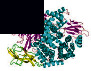	82	−705.7	−882.6
hAPN-mut1	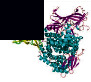	94	−849.3	−849.3
hAPN-mut2	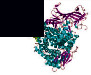	91	−833.1	−833.1
hAPN-mut3	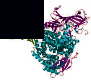	87	−852.3	−852.3
hAPN-mut4	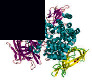	95	−597.1	−813.6
hAPN-mut5	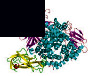	75	−657.9	−757.4
hAPN-mut6	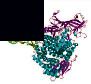	86	−849.0	−849.0
hAPN-mut7	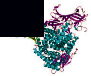	85	−836.6	−836.6
hAPN-mut8	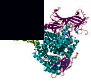	119	−887.3	−887.3
hAPN-mut9	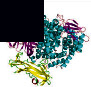	66	−720.7	−734.9
hAPN-mut10	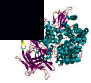	61	−585.1	−679.6
hAPN-mut11	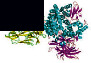	103	−718.2	−801.4

**Table 3:** Prediction of protein–protein interactions for manipulated mut8 set using ClusPro 2.0 tool.

Proteins	Structure	Members	Weighted score center	Weighted score lowest energy
hAPN-mut8_1	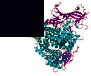	96	−844.4	−844.4
hAPN-mut8_2	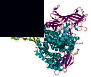	90	−849.4	−849.4
hAPN-mut8_3	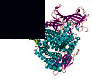	100	−851.6	−851.6
hAPN-mut8_4	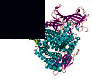	112	−863.4	−863.4

**Table 4:** Probabilities of mutations per spike gene with respect to different mutation rate values for coronaviruses.

Parameters	Per year^a^	Per transmission^b^
Mutation rate	1 × 10^−3^ substitution/base	1 × 10^−4^–1 × 10^−5^ substitution/base
Mutations per genome (∼30,000 base)	30 nucleotides	3–0.3 nucleotides
Mutations per spike gene (∼4500)	0.45 nucleotides	4.5 × 10^−2^–4.5 × 10^−3^ nucleotides

^a^Mutation rate under neutral genetic drift conditions estimated by Dorp et al. [52].

^b^Mutation rate per transmission reported by Van Egeren et al. [53].

## Data Availability

The data that supports the findings of this study are available in the supporting information of this article.

## Supporting Information

Supporting Information 1 File S1: Amino acid sequences of the proteins used for modeling.

Supporting Information 2 File S2: Protein–protein BLAST results.

Supporting Information 3 File S3: Prediction of protein–protein interactions using CCharPPI Server.
