# Supplementary material for: In Silico Assessment for Risk of Possible Human Transmission of FCoV-23
Source: Transbound Emerg Dis. 2024 Oct 1;2024:8398470. doi: 10.1155/2024/8398470 (PMC12017019; doi:10.1155/2024/8398470)
Supplement: Supporting Information 1 — File S1: Amino acid sequences of the proteins used for modeling. [file 8398470.f1.docx]

1. Amino acid sequence of feline APN protein:

>fAPN

MAKGFYISKPVGILAILLGVAAVCTIIALSVVYSQEKNRSTESSTAASTAAPTGPTTTVATTLDQSKPWNVYRLPKTLIPDSYNVTLRPYLTPNNKGLYVFTGTNIVRFTCKESTNIVIIHSKRLNYTSHQGHMVALSGVGGFHPQPVIVRTELVELTEYLVVHLQEPLVAGRQYEMNSEFQGELADDLAGFYRSEYMENGVKKVLATTHMQATEARKSFPCFDEPAMKATFNITIIHPNNLVALSNMLPRGPSVPFGEDPTWKVTEFETTPIMSTYLLAYIVSEFSYVETRAPSGVLIRIWARPSAINQGHGDYALKVTGPILDFFSQHYDTPYPLNKSDQIALPDFNAGAMENWGLVTYRESALLYDRQSSSSGNQERVVTVIAHELAHQWFGNLVTLEWWNDLWLNEGFASYVEYLGADFAEPTWNLKDLMVLNDVYRVMAVDALASSHPLSTPASEINTPAQISEVFDSISYSKGASVLRMLSNFLTEDLFKMGIASYLHTYKYGNTIYLNLWEHLQQVVDKQPTIKLPDTVSAIMDRWILQMGFPVITVDTQTGTISQQHFLLDPQSVVTRPSQFNYLWIVPISSVRSGSPQAHYWLPGVEKAQNDLFKTTANDWVLLNLNVTGYYLVNYDNENWKKIQTQLQTDLSVIPVINRAQVIHDAFNLASAQKVPVTLALNNTLFLIQETEYMPWQAALSSLSYFKLMFDRSEVYGPMKRYLKKQVTPLFNHFERVTKNWTDHPQTLMDQYSEINAVSTACSYGVPECEKLAATLFAQWKKNPQNNPIHPNLRSTVYCNAIAQGGEEEWNFVWEQFLKAELVNEADKLRGALACSNQVWILNRFLSYTLDPNLIRKQDVTSTLSSISSNVVGQTLVWDFVQSNWKKLFQDYGTGSFSFSNLIQAVTRRFSTEFELQQLEQFKKNNMDTGFGSATRALEQALEKTKANLKWVKENKDVVLRWFTENS

2. Amino acid sequence of human APN protein:

>hAPN

MAKGFYISKSLGILGILLGVAAVCTIIALSVVYSQEKNKNANSSPVASTTPSASATTNPASATTLDQSKAWNRYRLPNTLKPDSYRVTLRPYLTPNDRGLYVFKGSSTVRFTCKEATDVIIIHSKKLNYTLSQGHRVVLRGVGGSQPPDIDKTELVEPTEYLVVHLKGSLVKDSQYEMDSEFEGELADDLAGFYRSEYMEGNVRKVVATTQMQAADARKSFPCFDEPAMKAEFNITLIHPKDLTALSNMLPKGPSTPLPEDPNWNVTEFHTTPKMSTYLLAFIVSEFDYVEKQASNGVLIRIWARPSAIAAGHGDYALNVTGPILNFFAGHYDTPYPLPKSDQIGLPDFNAGAMENWGLVTYRENSLLFDPLSSSSSNKERVVTVIAHELAHQWFGNLVTIEWWNDLWLNEGFASYVEYLGADYAEPTWNLKDLMVLNDVYRVMAVDALASSHPLSTPASEINTPAQISELFDAISYSKGASVLRMLSSFLSEDVFKQGLASYLHTFAYQNTIYLNLWDHLQEAVNNRSIQLPTTVRDIMNRWTLQMGFPVITVDTSTGTLSQEHFLLDPDSNVTRPSEFNYVWIVPITSIRDGRQQQDYWLIDVRAQNDLFSTSGNEWVLLNLNVTGYYRVNYDEENWRKIQTQLQRDHSAIPVINRAQIINDAFNLASAHKVPVTLALNNTLFLIEERQYMPWEAALSSLSYFKLMFDRSEVYGPMKNYLKKQVTPLFIHFRNNTNNWREIPENLMDQYSEVNAISTACSNGVPECEEMVSGLFKQWMENPNNNPIHPNLRSTVYCNAIAQGGEEEWDFAWEQFRNATLVNEADKLRAALACSKELWILNRYLSYTLNPDLIRKQDATSTIISITNNVIGQGLVWDFVQSNWKKLFNDYGGGSFSFSNLIQAVTRRFSTEYELQQLEQFKKDNEETGFGSGTRALEQALEKTKANIKWVKENKEVVLQWFTENSK

3. Amino acid sequence of FCoV-23 RBD:

>FCoV-23 RBD

YCTGYATNVFAPTGGYIPDGFSFNNWFLLTNDSTFVSGRFVTNQPLLVNCLWPVPSFGVAAQEFCFEGAQFSQCNGVSLNNTVDVIRFNLNFTADVQSGMGATVFSLNTTGGVILEISCYNDTVRESSFYSYGEIPFGITDGPKYCYVLYNGTALKYLGTLPPSVKEIAISKWGHFYINGYNFFSTFPIDCISFNLTTSTSGAFWTIAYTSYTEALVQVENTAIKKVTYCNSHINNIKCSQLTANLQNGFYPVASSEVGLVNKSVVLLPSFYSHTSVNITIDLGMKLSGYGQPIASALSNITLPMQDNNTDVYCIRSNQFSVYVHSTCKSSLWDNVFNSDCTDVLHATAVIKTGTCPFSFDKLNNYLTFNKFCLSLHPVGANCKFDVAARTRTNEQVVRSLYVIYEEGDNIAGVPS
