# Supplementary material for: In Silico Assessment for Risk of Possible Human Transmission of FCoV-23
Source: Transbound Emerg Dis. 2024 Oct 1;2024:8398470. doi: 10.1155/2024/8398470 (PMC12017019; doi:10.1155/2024/8398470)
Supplement: Supporting Information 2 — File S2: Protein–protein BLAST results. [file 8398470.f2.docx]

Protein-protein BLAST results.


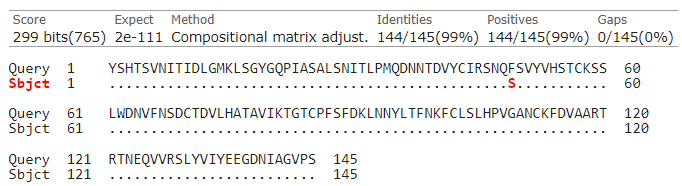


**Figure S1.** BLAST result of randomly created singular mutation (mut1).


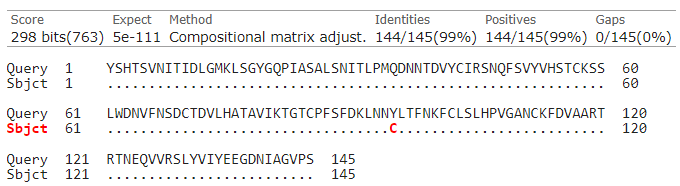


**Figure S2.** BLAST result of randomly created singular mutation (mut2).


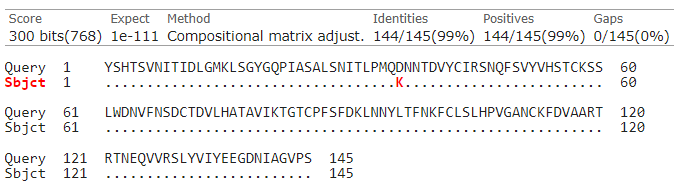


**Figure S3.** BLAST result of randomly created singular mutation (mut3).


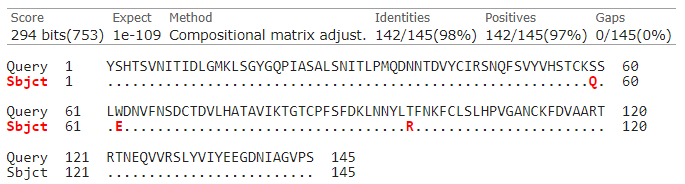


**Figure S4.** BLAST result of randomly created triple mutations (mut4).


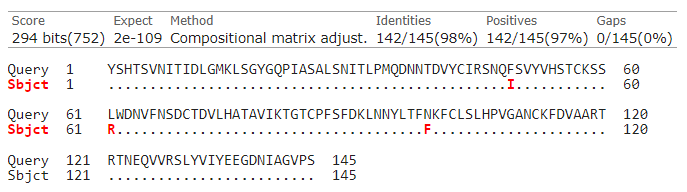


**Figure S5.** BLAST result of randomly created triple mutations (mut5).


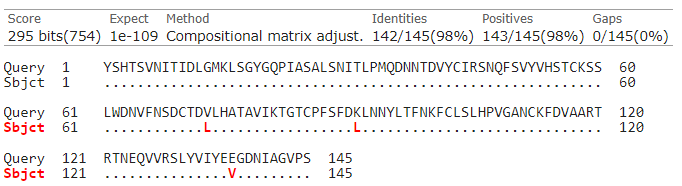


**Figure S6.** BLAST result of randomly created triple mutations (mut6).


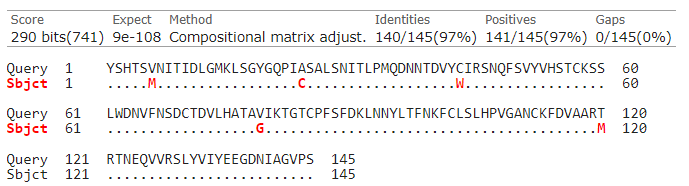


**Figure S7.** BLAST result of randomly created quinary mutations (mut7).


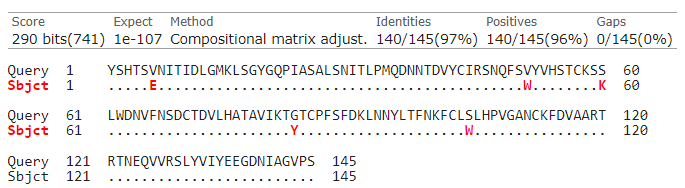


**Figure S8.** BLAST result of randomly created quinary mutations (mut8).


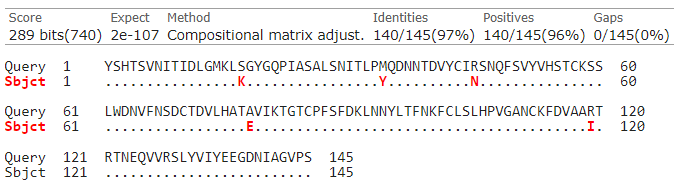


**Figure S9.** BLAST result of randomly created quinary mutations (mut9).


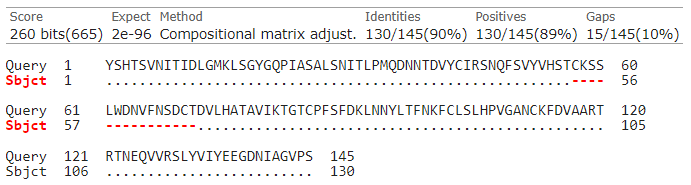


**Figure S10.** BLAST result of rationally created mutations (mut10).


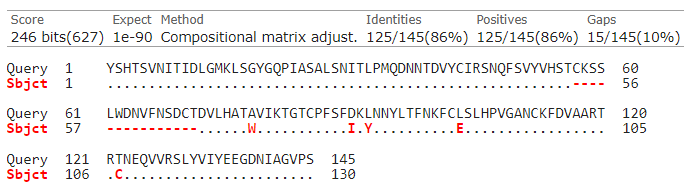


**Figure S11.** BLAST result of rationally created mutations (mut11).


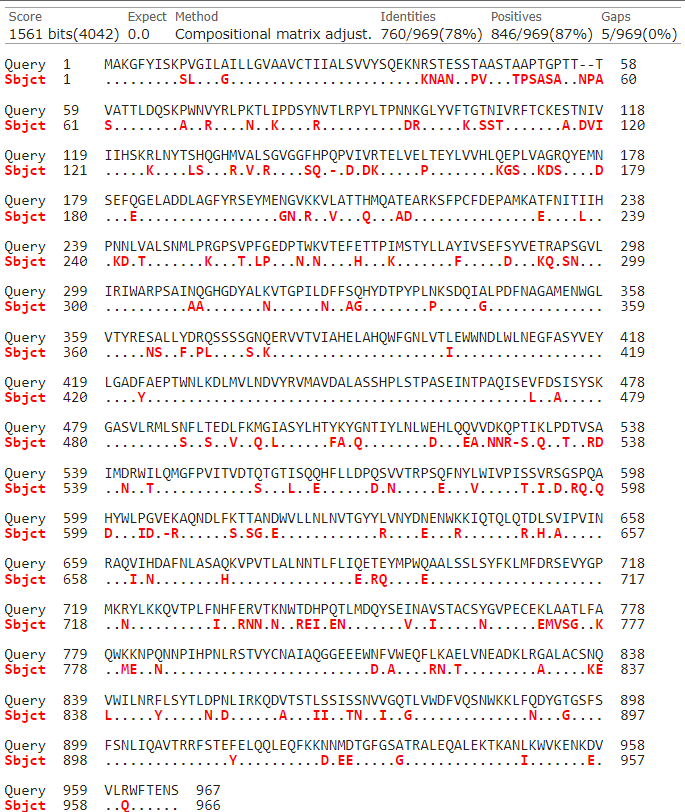


**Figure S12.** BLAST result of fAPN and hAPN proteins.


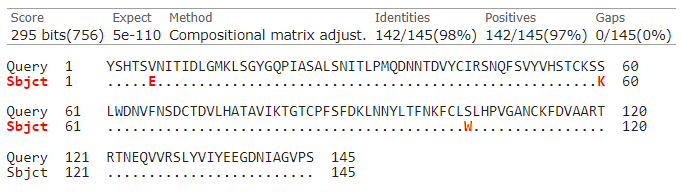


**Figure S14.** BLAST result of further created mutations on mut8 (mut8_1).


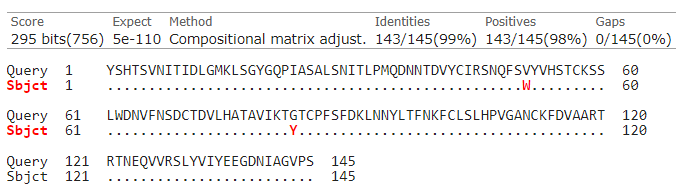


**Figure S15.** BLAST result of further created mutations on mut8 (mut8_2).


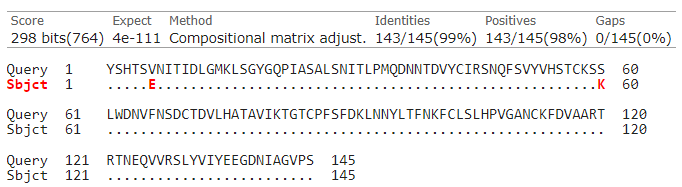


**Figure S16.** BLAST result of further created mutations on mut8 (mut8_3).


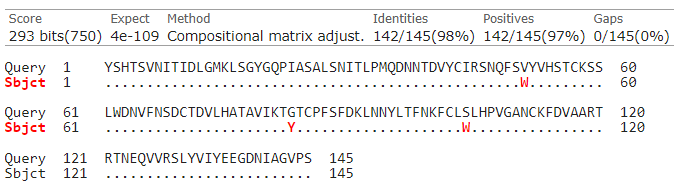


**Figure S17.** BLAST result of further created mutations on mut8 (mut8_4).
