# Supplementary material for: In Silico Assessment for Risk of Possible Human Transmission of FCoV-23
Source: Transbound Emerg Dis. 2024 Oct 1;2024:8398470. doi: 10.1155/2024/8398470 (PMC12017019; doi:10.1155/2024/8398470)
Supplement: Supporting Information 3 — File S3: Prediction of protein–protein interactions using CCharPPI Server. [file 8398470.f3.docx]

**Table S1.** Prediction of protein-protein interactions using CCharPPI Server.

| **Proteins** | **AP_DComplex** | **ZRANK** | **RosettaDock** | **pyDock** |
| --- | --- | --- | --- | --- |
| hAPN-mut1 | -4.7 | -0.22 | 0.009 | 0.41 |
| hAPN-mut2 | -4.7 | -0.22 | 0.009 | 0.42 |
| hAPN-mut3 | -4.7 | -0.22 | 0.008 | -0.39 |
| hAPN-mut4 | -4.7 | 6.75 | 0.03 | 3.91 |
| hAPN-mut5 | -4.7 | -1.11 | 0.008 | -1.10 |
| hAPN-mut6 | -4.7 | -0.22 | 0.008 | 0.49 |
| hAPN-mut7 | -4.7 | -0.22 | 0.008 | 0.41 |
| hAPN-mut8 | -4.7 | -0.70 | 0.008 | -0.06 |
| hAPN-mut9 | -4.7 | -0.70 | 0.008 | -0.06 |
| hAPN-mut10 | -4.7 | -1.22 | 0.008 | -1.34 |
| hAPN-mut11 | -4.7 | 0 | 0 | - |


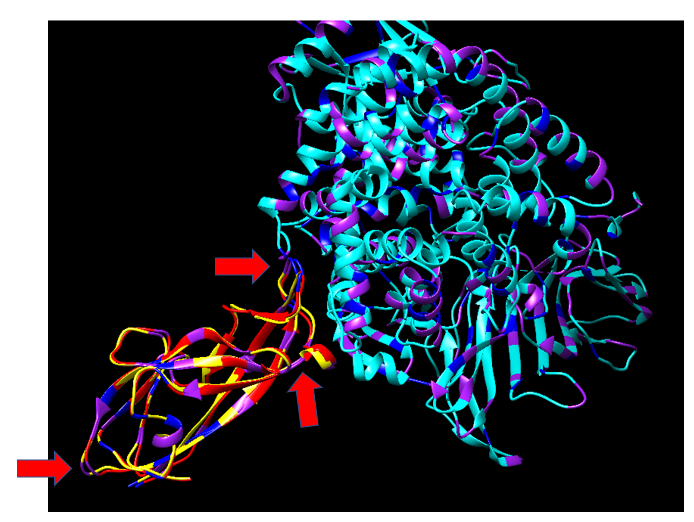


**Figure S13.** Altered structure by mutations (mut8) compared to the wild type RBD. Red arrows show the differentiated regions on β-sheets.
